# Association of anti-diabetic drugs and COVID-19 outcomes in patients with diabetes mellitus type 2 and cardiomyopathy

**DOI:** 10.1038/s41598-024-57871-9

**Published:** 2024-03-27

**Authors:** Jelena Dimnjaković, Tamara Buble, Pero Ivanko, Ivan Pristaš, Ognjen Brborović, Hana Brborović

**Affiliations:** 1 Division for Health Informatics and Biostatistics, Croatian Institute of Public Health, Rockefeller’s Street 7, 10 000 Zagreb, Croatia; 2 School of Medicine, Andrija Štampar School of Public Health, Department of Social Medicine and Health Care Organization, University of Zagreb, Rockefeller’s Street 4, 10 000 Zagreb, Croatia; 3 School of Medicine, Andrija Štampar School of Public Health, Department of Environmental and Occupational Health and Sports Medicine, University of Zagreb, Rockefeller’s Street 4, 10 000 Zagreb, Croatia

**Keywords:** Diabetes mellitus type 2, COVID-19, Hypoglycemic agents, Sodium-glucose transporter 2 inhibitors, Insulin, Dipeptidyl-peptidase 4 inhibitors, Repaglinide, Sulfonylurea compounds, Metformin, Pioglitazone, Acarbose, Diabetes, Cardiomyopathies, Viral infection

## Abstract

There is a scarcity of information on the population with diabetes mellitus type 2 and cardiomyopathy (PDMC) in COVID-19, especially on the association between anti-diabetic medications and COVID-19 outcomes. Study is designed as a retrospective cohort analysis covering 2020 and 2021. Data from National Diabetes Registry (CroDiab) were linked to hospital data, primary healthcare data, the SARS-CoV-2 vaccination database, and the SARS-CoV-2 test results database. Study outcomes were cumulative incidence of SARS-CoV-2 positivity, COVID-19 hospitalizations, and COVID-19 deaths. For outcome predictors, logistic regression models were developed. Of 231 796 patients with diabetes mellitus type 2 in the database, 14 485 patients had cardiomyopathy. The two2-year cumulative incidence of all three studies' COVID-19 outcomes was higher in PDMC than in the general diabetes population (positivity 15.3% vs. 14.6%, p = 0.01; hospitalization 7.8% vs. 4.4%, p < 0.001; death 2.6% vs. 1.2%, p < 0.001). Sodium-Glucose Transporter 2 (SGLT-2) inhibitors therapy was found to be protective of SARS-CoV-2 infections [OR 0.722 (95% CI 0.610–0.856)] and COVID-19 hospitalizations [OR 0.555 (95% CI 0.418–0.737)], sulfonylureas to be risk factors for hospitalization [OR 1.184 (95% CI 1.029–1.362)] and insulin to be a risk factor for hospitalization [OR 1.261 (95% CI 1.046–1.520)] and death [OR 1.431 (95% CI 1.080–1.897)]. PDMC are at greater risk of acquiring SARS-CoV-2 infection and having worse outcomes than the general diabetic population. SGLT-2 inhibitors therapy was a protective factor against SARS-CoV-2 infection and against COVID-19 hospitalization, sulfonylurea was the COVID-19 hospitalization risk factor, while insulin was a risk factor for all outcomes. Further research is needed in this diabetes sub-population.

## Introduction

Since the beginning of the Corona Virus Disease-19 (COVID-19) pandemic, clinicians and researchers have worried that anti-diabetic medications may lead to worse COVID-19 outcomes and increased SARS-CoV-2 infections in patients using these medications. The fear partly rests in the fact that the receptor for SARS-CoV-2, angiotensin-converting enzyme-2 (ACE-2), is possibly overexpressed during the use of some of these medications^1^. Key opinion leaders in diabetes management have recommended continuing the usual anti-diabetic treatment until further evidence is gathered, calling for research^2,3^.

Patients suffering from diabetes mellitus type 2, even before the above-mentioned anti-diabetic medications andCOVID-19-issue, already had reasons to be worried since the diabetes population has experienced increased rates of SARS-CoV-2 infections, COVID-19 hospitalizations, and deaths in comparison to the general population^4,5^. Diabetes patients with comorbidities such as cardiovascular disease, nephropathy, and cardiomyopathy have been even more prone to the horrors of SARS-CoV-2 than "ordinary" diabetes patients^4,5^.

Therefore, we have decided to analyse the entire diabetes population and association between anti-diabetic drugs and COVID-19 outcomes, but while conducting the research and reading the literature, we could not help but wander about subpopulation of diabetes patients with cardiomyopathy. This particular study focuses on patients with diabetes mellitus type 2 and cardiomyopathy (PDMC).

We focused on cardiomyopathy due to the interesting notion of the so-called "diabetic cardiomyopathy". This is cardiac dysfunction in patients with diabetes who do not necessarily show any signs of coronary artery disease or other usual risk factors for cardiomyopathy development^5^. It happens due to disrupted glucose and fatty acid metabolisms^6^. It can lead to heart failure, which is associated with increased mortality and poor COVID-19 prognosis^7–9^.

We found no published data on how this sub-population of patients with diabetes has fared during the COVID-19 pandemic regarding COVID-19 outcomes and if and how anti-diabetic medications are associated with COVID-19 outcomes in these patients.

Diabetes key opinion leaders also seem interested to know more about this population and COVID-19^4,5^. While there are published studies about the association of anti-diabetic medications and SARS-CoV-2 infection and COVID-19 outcomes in a population of patients with diabetes, we found no published clinical studies explicitly conducted on PDMC.

This means that these people and their healthcare providers benefit from research that sheds some light on anti-diabetic medications and COVID-19 issues. In our study, we wanted to:

1. Evaluate the prevalence of cardiomyopathy in patients with diabetes mellitus type 2,
2. Evaluate the 2-year cumulative incidence (years 2020 and 2021) of SARS-CoV-2 infections, COVID-19 hospitalizations, and COVID-19 deaths among PDMC,
3. Describe differences in the incidence of SARS-CoV-2 infections, COVID-19 hospitalizations, and deaths in different groups depending on the type of anti-diabetic therapy, and
4. Analyze risk factors for SARS-CoV-2 infection, COVID-19 hospitalization, and COVID-19 death in the observed population while focusing on anti-diabetic therapy.

## Methodology

The study was a retrospective data analysis covering the period from Jan 1st 2020 to Dec 31st 2021. Characteristics of the entire population of patients with diabetes mellitus type 2 in Croatia were analyzed, focusing on the sub-population of people with cardiomyopathy—PDMC.

Croatian National Diabetes Registry (CroDiab) was the source of data. CroDiab contains individual longitudinal data on patients with diabetes mellitus^10,11^. Several sources are being used to feed CroDiab with data via the National Public Health Information System of Croatia and the Central Health Information System of the Republic of Croatia: clinical laboratories, primary health care providers, and hospitals^12,13^. For our study, CroDiab was linked to a database containing SARS-CoV-2 test results, the National Vaccination Database (eVac), and the National Causes of Death Registry using a common personal identifier^14,15^. The resulting data export was anonymized.

The outcome of SARS-CoV-2 infection was defined as the first or only positive test result (nasopharyngeal swab, Polymerase Chain Reaction (PCR)). According to hospital data, COVID-19 hospitalization outcome was defined as a hospitalization with COVID-19 being the primary or secondary diagnosis described. COVID-19 death outcome was defined as death, with COVID-19 listed as the primary source of death per the National Causes of Death Registry. The diagnosis of COVID-19 was determined per the World Health Organization International Statistical Classification of Diseases and Related Health Problems, 10th revision (ICD-10), code U07. All COVID-19 diagnoses were laboratory-confirmed by PCR test.

Anti-diabetic drug intake was defined if a prescription was picked up at least two times in eight months before the SARS-CoV-2 or COVID-19 outcome. If the person experienced none of the outcomes, therapy was defined if a prescription was picked-up up at least once eight months before the patient visited her primary healthcare provider with a diagnosis of diabetes mellitus recorded in the system during that visit. Glycated hemoglobin (HbA1C) and body mass index (BMI) data were searched six months before outcomes or primary health care visits.

For CroDiab purposes, a person is classified as a person with diabetes mellitus if at least one of the following conditions are met: (1) at least one hospital report with diabetes mellitus diagnosis was found in the system, (2) if the person visited her primary healthcare provider at least twice and ICD-10 diagnoses of E10–E14 were recorded during the visit, (3) if the person picked up at least two prescriptions with diagnoses E10–E14 or if the prescriptions had Anatomical Therapeutic Chemical Classification (ATC) codes A10 excluding code A10BA, (4) if person's primary healthcare provider reported the person as diabetes mellitus patient via the National Public Health Information System plus the person visited her primary healthcare provider at least once and ICD-10 diagnoses of E10–E14 were recorded during the visit or the person picked up at least one prescription with diagnoses E10-E14 or if the prescription had ATC codes A10 excluding code A10BA^16^.

Cardiomyopathy was defined as ICD-10 diagnosis I42 (Cardiomyopathy) recorded at least twice in the system from Jan 1st 2018 onwards.

Individual comorbidities were identified if their ICD-10 codes were recorded at least twice in the system from Jan 1st 2018 onwards. ICD-10 codes looked for were: malignant neoplasms (C00–C97); hypertensive diseases (I10–I15); ischemic heart diseases (I20–I25); cerebrovascular diseases (I60–I69); diseases of the circulatory system excluding hypertension (I00–I09 and I20–I99); chronic lower respiratory diseases (J40–J47); other chronic obstructive pulmonary disease (J44); chronic kidney disease (N18).

Inclusion criteria for data analysis were type 2 diabetes mellitus, defined as per CroDiab definition already described, and age of 18 years or more. The exclusion criteria were lack of reliable data on anti-diabetic drug use. The latter patients were omitted from the analysis.

The Croatian Institute of Public Health Ethical Committee and the University of Zagreb Medical School Ethical Committee approved the study. Need for informed consent was waived by The Croatian Institute of Public Health Ethical Committee. The study has been performed in accordance with the Declaration of Helsinki.

### Statistical analysis

Differences between groups of independent continuous variables were analyzed with the t-test, whereas differences in the prevalence of individual conditions were compared with the χ^2^ test. The level of significance was set at α = 0.05.

Logistic regression analysis was used to determine the relative risks of developing outcomes. Univariate regression models were performed with each of the variables. Only the variables with a statistically significant association in the univariate logistic regression model, i.e., those with 95% confidence intervals (CI) not including 1, were included in the multiple logistic regression model. In the multiple models, Odds ratios (OR) and 95% CI were determined.

In our initial statistical analysis plan, these covariates were systematically forced into the models: age, sex, BMI and HbA1c, diabetes mellitus duration, ACE inhibitors (ACEI) or angiotensin receptor blockers (ARB) intake, SARS-CoV-2 vaccination data, comorbidities, and anti-diabetic drugs. However, models with BMI could not perform due to the deficient number of BMI data available, so this variable was not included in further models. Since HbA1c did not contribute to the risk of any of the outcomes, and owing to a significant number of missing data for HbA1c, our univariate and multivariable models ultimately did not take HbA1c into account, neither BMI.

Analyses were performed with IBM SPSS Statistics software, version 29.0 (IBM, Armonk, NY, USA).

### Ethics approval and consent to participate

The Croatian Institute of Public Health Ethical Committee and the University of Zagreb Medical School Ethical Committee approved the study. Need for informed consent was waived by The Croatian Institute of Public Health Ethical Committee.

## Results

There were 310,749 patients with diabetes in the CroDiab database older than 18 years of age and with diabetes mellitus type 2. After removing patients without reliable anti-diabetic therapy data (N = 78,953), we were left with 231,796 patients. Out of these, 14,485 had cardiomyopathy. Table 1 shows the demography and characteristics of these patients.

**Table 1:** Characteristics of patients with diabetes mellitus type 2 and cardiomyopathy (N = 14,485).

Demography	
Male sex, N (%)	7712 (53.2%)
Age in years, mean ± SD	73.9 ± 10.0
Diabetes duration in years, mean ± SD	6.5 ± 4.0
COVID-19 outcomes	
SARS-Cov-2 positive, N (%)	2221 (15.3%)
COVID-19 hospitalized, N (%)	1136 (7.8%)
COVID-19 deaths, N (%)	370 (2.6%)
ACEI or ARB	
Yes, N (%)	10,318 (71.2%)
SARS-CoV-2 vaccination	
Dose 1, N (%)	9187 (63.4%)
Dose 2, N (%)	8594 (59.3%)
Booster, N (%)	4020 (27.8%)
Comorbidities other than cardiomyopathy	
Diseases of the circulatory system excluding hypertension, N (%)	14,485 (100%)
Hypertensive diseases, N (%)	12,728 (87.9%)
Ischaemic heart diseases, N (%)	4962 (34.3%)
Chronic lower respiratory diseases, N (%)	3436 (23.7%)
Other chronic obstructive pulmonary disease, N (%)	2354 (16.3%)
Cerebrovascular diseases, N (%)	1967 (13.6%)
Malignant neoplasms, N (%)	1761 (12.2%)
Chronic kidney disease, N (%)	1696 (11.7%)
Diabetes mellitus chronic treatment	
Biguanides (only metformin), N (%)	9672 (66.8%)
Sulfonylureas, N (%)	4953 (34.2%)
DPP-4 inhibitors, N (%)	3865 (26.7%)
Insulin, N (%)	2341 (16.2%)
SGLT-2 inhibitors, N (%)	1565 (10.8%)
GLP-1 analogues, N (%)	909 (6.3%)
Repaglinide, N (%)	744 (5.1%)
Thiazolidinediones (only pioglitazone), N (%)	552 (3.8%)
Alpha glucosidase inhibitors (only acarbosis), N (%)	90 (0.6%)
Diabetes characteristics	
Body mass index in kg/m^2^, mean ± SD*	30.4 ± 6.4
Glycated haemoglobin HbA1c in %, mean ± SD**	7.1 ± 1.7

*ACEI* angiotensin-converting enzyme inhibitor, *ARB* angiotensin receptor blockers, *DPP-4* Dipeptidyl peptidase 4, *SGLT-2* Sodium-glucose co-transporter 2, *GLP-1* Glucagon-like peptide-1.

*Available for 25 patients.

**Available for 259 patients.

PDMC were predominantly male, of old age, with a mean diabetes duration of almost 6.5 years. All of them have circulatory or hypertensive diseases, 1/3 have ischemic heart disease, and 1/3 have some chronic respiratory disease, slightly above 10% have chronic kidney disease. All of them are taking either metformin or one of the sulfonylureas. About 60% of them have received 1 or 2 doses of a SARS-CoV-2 vaccine and nearly a quarter a booster dose. Two-year cumulative incidence of SARS-CoV-2 infections was 15.3%, COVID-19 hospitalizations 7.8%, and COVID-19 deaths 2.6%.

COVID-19 incidence in PDMC is compared with the incidence entire diabetic population in Table 2.

**Table 2:** SARS-CoV-2 and COVID-19 epidemiology in studied cohort, comparison to the entire diabetes mellitus type 2 population.

Outcomes	PDMC (N = 14,485)	DM2 (N = 231,796 )	P
SARS-CoV-2 positivity, N (%)	2221 (15.3%)	33,741 (14.6%)	0.01
COVID-19 hospitalizations, N (%)	1136 (7.8%)	10,191 (4.4%)	< 0.001
COVID-19 deaths, N (%)	370 (2.6%)	2692 (1.2%)	< 0.001

2-year cumulative incidence presented (years 2020 and 2021);

P values calculated via hi square test;

*PDMC* Patients with diabetes mellitus type 2 and cardiomyopathy, *DM2* Entire diabetes mellitus type 2 population.

PDMC have fared significantly worse than the entire diabetes mellitus type 2 population during the pandemic, especially regarding hospitalizations and deaths, with twice as many deaths and 1.8 times higher hospitalizations incidence.

Differences in the incidence of SARS-CoV-2 infections, COVID-19 hospitalizations, and deaths in different PDMC groups depending on the type of anti-diabetic therapy are shown in Table 3.

**Table 3:** SARS-CoV-2/COVID-19 outcomes in patients with diabetes mellitus type 2 and cardiomyopathy depending on antidiabetic therapy.

Outcomes [presented as N (%)]	Antidiabetic medication		P
	SGLT-2 inhibitors yes N = 1565	SGLT-2 inhibitors no N = 12,920	
SARS-CoV-2 infections	188 (12.00%)	2033 (15.70%)	** < 0.001**
COVID-19 hospitalized	58 (3.70%)	1078 (8.30%)	** < 0.001**
COVID-19 death	17 (1.10%)	353 (2.70%)	** < 0.001**
	Metformin yes N = 9672	Metformin no N = 4813	
SARS-CoV-2 infections	1440 (14.90%)	781 (16.20%)	**0.035**
COVID-19 hospitalized	679 (7.00%)	457 (9.50%)	** < 0.001**
COVID-19 death	202 (2.10%)	168 (3.50%)	** < 0.001**
	Sulfonylureas yes N = 4953	Sulfonylureas no N = 9532	
SARS-CoV-2 infections	783 (15.80%)	1438 (15.10%)	0.253
COVID-19 hospitalized	455 (9.20%)	681 (7.10%)	** < 0.001**
COVID-19 death	144 (2.90%)	226 (2.40%)	0.058
	DPP-4 inhibitors yes N = 3865	DPP-4 inhibitors no N = 10,620	
SARS-CoV-2 infections	637 (16.50%)	1584 (14.90%)	**0.021**
COVID-19 hospitalized	324 (8.40%)	812 (7.60%)	0.151
COVID-19 death	107 (2.80%)	263 (2.50%)	0.341
	GLP-1 analogues yes N = 909	GLP-1 analogues no N = 13,576	
SARS-CoV-2 infections	142 (15.60%)	2079 (15.30%)	0.816
COVID-19 hospitalized	45 (5.00%)	1091 (8.00%)	** < 0.001**
COVID-19 death	14 (1.50%)	356 (2.60%)	0.055
	Acarbosis yes N = 52	Acarbosis no N = 7660	
SARS-CoV-2 infections	10 (11.10%)	2211 (15.40%)	0.306
COVID-19 hospitalized	4 (4.40%)	1132 (7.90%)	0.322
COVID-19 death	2 (2.20%)	368 (2.60%)	1
	Pioglitazone yes N = 552	Pioglitazone no N = 13,933	
SARS-CoV-2 infections	93 (16.80%)	2128 (15.30%)	0.307
COVID-19 hospitalized	42 (7.60%)	1094 (7.90%)	0.927
COVID-19 death	12 (2.20%)	358 (2.60%)	0.680
	Repaglinide yes N = 744	Repaglinide no N = 13,741	
SARS-CoV-2 infections	111 (14.90%)	2110 (15.40%)	0.790
COVID-19 hospitalized	67 (9.00%)	1069 (7.80%)	0.233
COVID-19 death	24 (3.20%)	346 (2.50%)	0.231
	Insulin yes N = 2341	Insulin no N = 12,144	
SARS-CoV-2 infections	408 (17.40%)	1813 (14.90%)	**0.002**
COVID-19 hospitalized	222 (9.50%)	914 (7.50%)	**0.001**
COVID-19 death	86 (3.70%)	284 (2.30%)	** < 0.001**

P calculated via hi square test.

Statistically significant p values are bolded.

Patient characteristics for each group are presented in Additional file 1.

*SGLT-2* Sodium-glucose Cotransporter-2, *DPP-4* Dipeptidyl Peptidase 4, *GLP-1* Glucagon-like peptide 1.

The SGLT-2 inhibitors and metformin group had a lower incidence of SARS-Cov-2 infections, COVID-19 hospitalizations, and COVID-19 deaths compared to SGLT-2 and metformin nonusers, respectively. Group using GLP-1 analogs had a lower incidence of hospitalization than nonusers. Group using sulfonylureas showed an increased incidence of hospitalizations than nonusers. Group using DPP-4 inhibitors had a higher incidence of infections. The insulin group showed an increased incidence of all three outcomes compared to nonusers.

Patient characteristics regarding demography, comorbidities, ACEI or ARBs intake, and SARS-CoV-2 vaccination status are presented in Additional file 1.

Tables 4, 5, and 6 show all three outcomes' final multiple regression models. Univariate models for each outcome are presented as Additional file 2.

**Table 4:** Final multiple regression model for outcome of SARS-CoV-2 infection.

Variable	p	Odds ratio	95% confidence interval
**Age in years**	** < 0.001**	**0.987**	**0.982–0.992**
**Female sex**	** < 0.001**	**0.803**	**0.728–0.886**
**Diabetes duration ≤ 2 years**	**0.002**	**0.670**	**0.522–0.860**
**Diabetes duration in years**	**0.040**	**1.014**	**1.001–1.027**
Insulin	0.259	1.080	0.945–1.234
**SARS-cov-2 vaccination dose 1**	** < 0.001**	**1.482**	**1.222–1.797**
**SARS-cov-2 vaccination dose 2**	** < 0.001**	**0.537**	**0.442–0.652**
**SARS-cov-2 vaccination booster**	** < 0.001**	**0.305**	**0.262–0.355**
**Hypertensive diseases**	** < 0.001**	**1.313**	**1.127–1.529**
**Chronic lower respiratory diseases**	**0.008**	**1.262**	**1.064–1.497**
Other chronic obstructive pulmonary disease	0.921	0.990	0.815–1.203
**Chronic kidney disease**	** < 0.001**	**1.304**	**1.135–1.499**
**SGLT-2 inhibitors**	** < 0.001**	**0.722**	**0.610–0.856**
DPP-4 inhibitors	0.090	1.094	0.986–1.213
Metformin	0.463	1.040	0.936–1.156

P of the multivariate model < 0.001.

Bolded text in table represents variables with statistically significant association to outcome; Univariate models are available in Additional file 2.

**Table 5:** Final multiple regression model for outcome of COVID-19 hospitalization.

Variables	p	Odds ratio	95% confidence interval
**Age in years**	**0.032**	**1.008**	**1.001–1.015**
**Female sex**	** < 0.001**	**0.651**	**0.570–0.744**
Diabetes duration ≤ 2 years	0.057	0.707	0.495–1.010
Diabetes duration in years	0.599	1.005	0.987–1.022
**Insulin**	**0.015**	**1.261**	**1.046–1.520**
SARS-cov-2 vaccination dose 1	0.264	1.151	0.899–1.474
**SARS-cov-2 vaccination dose 2**	** < 0.001**	**0.398**	**0.307–0.517**
**SARS-cov-2 vaccination booster**	** < 0.001**	**0.336**	**0.264–0.428**
Malignant neoplasms	0.156	1.140	0.951–1.366
**Hypertensive diseases**	** < 0.001**	**1.451**	**1.170–1.799**
Ischaemic heart diseases	0.054	1.138	0.998–1.297
Cerebrovascular diseases	0.275	1.099	0.927–1.303
**Chronic lower respiratory diseases**	** < 0.001**	**1.482**	**1.189–1.847**
Other chronic obstructive pulmonary disease	0.833	1.027	0.802–1.314
**Chronic kidney disease**	** < 0.001**	**1.360**	**1.140–1.623**
**SGLT-2 inhibitors**	** < 0.001**	**0.555**	**0.418–0.737**
**Sulfonylureas**	**0.018**	**1.184**	**1.029–1.362**
GLP-1 analogues	0.142	0.784	0.567–1.084
Metformin	0.743	1.024	0.887–1.183

P of the multivariate model < 0.001.

Bolded text in table represents variables with statistically significant association to outcome; Univariate models are available in Additional file 2.

**Table 6:** Final multiple regression model for outcome of COVID-19 death.

Variable	p	Odds ratio	95% confidence interval
**Age in years**	** < 0.001**	**1.023**	**1.010–1.036**
**Female sex**	** < 0.001**	**0.462**	**0.368–0.580**
Diabetes duration ≤ 2 years	0.180	0.619	0.307–1.249
Diabetes duration in years	0.488	1.010	0.982–1.039
**Insulin**	**0.013**	**1.431**	**1.080–1.897**
**SARS-cov-2 vaccination dose 1**	**0.010**	**0.531**	**0.328–0.857**
**SARS-cov-2 vaccination dose 2**	** < 0.001**	**0.226**	**0.127–0.402**
**SARS-cov-2 vaccination booster**	** < 0.001**	**0.031**	**0.004–0.229**
Ischemic heart diseases	0.165	1.170	0.938–1.460
Cerebrovascular diseases	0.656	1.066	0.804–1.414
**Chronic lower respiratory diseases**	**0.021**	**1.550**	**1.070–2.247**
Other chronic obstructive pulmonary disease	0.796	1.055	0.702–1.586
Chronic kidney disease	0.169	1.225	0.918–1.636
SGLT-2 inhibitors	0.136	0.674	0.401–1.132
GLP-1 analogues	0.938	1.023	0.575–1.822
Metformin	0.555	0.932	0.740–1.176

P of the multivariate model < 0.001.

Bolded text in table represents variables with statistically significant association to outcome; Univariate models are available in Additional file 2.

In multiple regression models, insulin was the only anti-diabetic medication found to be associated with death outcomes (OR 1.431 (95% CI 1.080–1.897)). Other drugs showed no significant association with death in regression models. When it comes to COVID-19 hospitalization, SGLT-2 inhibitors were found to be a protective factor against hospitalization (OR 0.555 (95% CI 0.418–0.737)), while sulfonylurea and insulin were found to be risk factors for hospitalization (OR 1.184 (95% CI 1.029–1.362)) and (OR 1.261 (95% CI 1.046–1.520), respectively]. Other drugs showed no association with hospitalization outcomes. And lastly, regarding SARS-CoV-2 positivity, SGLT-2 inhibitors were found to be a protective factor (OR 0.722 (95% CI 0.610–0.856)) while no other drug showed an association.

## Discussion

Our study filled in the data gap on the subpopulation of patients suffering from diabetes mellitus type 2 with cardiomyopathy.

We showed these patients comprise 6.25% of diabetes-population listed in the CroDiab registry, that they are old (mean age ± SD 73.89 ± 9.97) years, predominantly male (53.2%), and that their diabetes duration was 6.49 ± 4.04 years (mean ± SD). All of our patients had "other circulatory disorders," almost 90% had arterial hypertension, and all were taking metformin or one of the sulfonylureas.

Our study showed that PDMC have fared significantly worse than the entire diabetes mellitus type 2 population during the pandemic, especially regarding hospitalizations and deaths, with twice as many deaths and 1.8 times higher hospitalizations incidence. These results are not surprising since it has been shown that diabetes mellitus and cardiomyopathy separately were predictors of SARS-CoV-2 infections, COVID-19 hospitalizations, and deaths^17^.

Not speaking of medications yet, but of other variables forced into our regression models (age, sex, diabetes duration, ACEI or ARB use, comorbidities, and SARS-CoV-2 vaccines), results of our models are in line with data in patients with diabetes; older people, men, with longer diabetes duration, not vaccinated are under greater risk of being hospitalized and dying^17^.

When it comes to anti-diabetic medications, our study showed that SGLT-2 inhibitors were associated with decreased risk of SARS-CoV-2 infections and COVID-19 hospitalizations, that insulin and sulphonylurea were associated with increased risk of COVID-19 hospitalizations and that insulin was associated with increased risk of COVID-19 death.

Since the beginning of the pandemic, clinicians and researchers have been scared that SGLT-2 inhibitors, GLP-1 receptor agonists, pioglitazone, and insulin might lead to overexpression of ACE-2 receptor and thus cause more SARS-Cov-2 infections and worse COVID-19 outcomes. However, there has also been awareness of the potential benefits of these drugs on COVID-19 outcomes. E.g., the benefit of both GLP-1 receptor agonists and SGLT-2 inhibitors in the prevention of cardiovascular and kidney disease^18^. Or the fact that pioglitazone, DPP-4 inhibitors, SGLT-2 inhibitors, GLP-1 receptor agonists, and insulin have shown anti-inflammatory activity, which could be very helpful during COVID-19^19^. Also, some anti-diabetic medications, such as SGLT-2 inhibitors, tend to lower the risk of heart failure in diabetic patients as a class effect^5^.

Diabetes key opinion leaders have recommended continuing anti-diabetic therapy until more is learned while waiting for clinical data^2^.

Our study researched outcomes of SARS-CoV-2 infection, COVID-19 hospitalization, and COVID-19 death in PDMC. During the literature search, we found no studies regarding the specific cardiomyopathy sub-population and COVID-19 outcomes, so we can only compare our data to studies conducted in the general diabetes population. Even though many of these studies have no comorbidities data, they are still useful for our targeted population as they give us a grasp of the bigger picture.

Our study showed insulin was a risk factor for COVID-19 hospitalization and death outcomes. This is following previous research on the population of patients with diabetes mellitus type 2. E.g., a meta-analysis found 2.2 times higher odds of death in patients using insulin vs. patients not-using insulin^20^. This was also confirmed in another meta-analysis^21^. Although the underlying mechanism is unclear, all these studies’ results suggest the need for careful assessment of the benefits and potential adverse effects of insulin therapy for patients with COVID-19^20^. It should be noted that in all cited studies insulin was shown to be independent predictor of mortality regardless of the age, sex, diabetes duration and gylcemic status^20,21^.

In our study, sulfonylurea was shown to be a predictor of COVID-19 hospitalization and showed no association with COVID-19 death outcome or SARS-CoV-2 positivity. Published observational trials conducted in patients with diabetes mellitus type 2 and COVID-19 and most meta-analyses also found no association with death outcomes, although, e.g., Kan et al. did find an association with lower mortality risk (pooled OR, 0.80; P = 0.016) in their meta-analysis^20–28^. One study suggested a borderline increased risk of adverse outcomes during hospitalization^29^. The specific underlying mechanism which would explain association between sufphonylurea and COVID-19 outcomes is unclear^20^. If islet function is acceptable, sulfonylurea drugs can be considered for hypoglycemic treatment in patients with diabetes mellitus type 2 who have COVID-19. However, sulfonylurea drugs can easily cause hypoglycemia; therefore, the use of sulfonylurea drugs in patients with severe COVID-19 requires careful blood glucose monitoring^20^.

Our study did not identify metformin, DDP-4 inhibitors, repaglinide, thiazolidinedione pioglitazone, GLP-1 receptor agonists, or alpha-glucosidase inhibitor acarbose to be associated with any of the study outcomes.

In a meta-analysis, thiazolidinedione (we studied pioglitazone), and alpha-glucosidase inhibitor (we looked at acarbose) were also found to be mortality neutral in patients with diabetes mellitus type 2 and COVID-19^27^.

GLP-1 receptor agonists show a protective association against COVID-19 mortality in patients with diabetes mellitus type 2 in meta-analyses^21,30^. Nassar et al. showed GLP-1 receptor agonists showed to be protective against COVID-19 hospitalization^30^.

We found several large meta-analyses regarding metformin's protective association against COVID-19 death and hospitalization risk^20,30,31^.

In literature, DPP-4 inhibitors show mixed results regarding the association with COVID-19 death and hospitalization in diabetes mellitus patients. Meta-analyses found an association with mortality reduction^21,32^. However, another meta-analysis found DPP-4 inhibitors use was associated with almost 1.5 times higher hospitalization risk and increased risk of ICU admissions and/or mechanical ventilation vs. nonusers^30^.

SGLT-2 inhibitors caught our eye by being presented as protective against SARS-CoV-2 infection and COVID-19 hospitalization by our regression models.

During the early months of the COVID-19 pandemic, some papers recommended that SGLT‐2 inhibitors be temporarily discontinued in hospitalized patients with diabetes mellitus^33,34^. These suggestions were based on mechanistic explanations. One explanation was that dehydration during acute illness (including COVID‐19) could predispose to lactic acidosis and diabetic ketoacidosis, and thus metformin and SGLT-2 inhibitors should be temporarily discontinued in hospitalized patients^33^. Another paper suggested discontinuation of SGLT-2 inhibitors in patients with diabetes and COVID-19 and avoidance of adding SGLT-2 inhibitors in anti-diabetic therapy for all patients with diabetes during the COVID-19 pandemic due to increased expression of ACE-2 enzyme, which could be an entry point for SARS-CoV-2^34^.

However, as clinical data started pouring in in the form of observational trials and meta-analyses, it was shown that probably no such precautions were necessary. Quite on the contrary, some papers suggested SGLT-2 inhibitors as drugs of choice^35^.

A meta-analysis and meta-regression conducted specifically to assess SGLT-2 inhibitors in diabetes patients with COVID-19 of a total of 17 studies showed that preadmission use of SGLT-2 inhibitors was associated with reduced mortality and severity of COVID-19. This benefit of SGLT-2 inhibitors on COVID-19 mortality was not significantly affected by patient factors such as age, sex, hypertension, heart failure, HbA1c levels, metformin use, duration of diabetes, and BMI. The paper's authors suggested SGLT-2 inhibitors could be considered an anti-diabetic drug of choice, especially during the pandemic^35^. Another, Bayesian meta-analysis of 35 studies on several anti-diabetic agents found that SGLT-2 inhibitors could reduce COVID-19 mortality risk in individuals with diabetes^34^.

Meta-analysis of 26 studies found a statistically significant decrease in hospitalization for SGLT-2 inhibitors users vs. nonusers (RR 0.89, 95% CI 0.84–0.95, p < 0.001), but no statistically significant effect of SGLT‐2 inhibitors use as regards intensive care unit (ICU) admission/mechanical ventilation and mortality^30^. The latter was confirmed in a randomized controlled trial comparing SGLT-2 inhibitor dapagliflozin to placebo among 1250 persons hospitalized with COVID‐19 and with at least one cardiometabolic risk factor (i.e., hypertension, type 2 diabetes, atherosclerotic cardiovascular disease, heart failure, and chronic kidney disease). The study found no statistically significant risk reduction in organ dysfunction or improvement in clinical recovery for patients using an SGLT-2 inhibitor, dapagliflozin, compared to a placebo^36^. It also found no significant risk reduction in death outcomes. Two studies revealed an association of decreased incidence of hospitalization in the SGLT‐2 inhibitors group compared with the DPP-4 inhibitors user group^37,38^. All this is far from the initial fear of SGLT-2 inhibitors use during COVID-19.

Our study found no association between SGLT-2 inhibitors and the death outcome. This does not align with some of the described meta-analyses showing SGLT-2 inhibitors as protective factors against COVID-19 death in the diabetes population^34,35^.

The outcomes of SARS-CoV-2 infection and COVID-19 hospitalization should not be underestimated. Recent evidence shows that SARS-CoV-2 sequels do not end when one survives or is no longer PCR positive but can continue in post-COVID-19 syndrome. Patients with diabetes mellitus type 2 and cardiac disorders are more prone to developing post-COVID-19 syndrome, especially if older and with multiple medical conditions, compared to the general population^39,40^. Therefore, preventing SARS-CoV-2 infection is vital in the context of retaining a level of quality of life and preventing serious illnesses which are known to be part of post-COVID-19 syndrome.

Additionally, as it has been known for a long time, hospitalization per se can be dangerous for patients with diabetes due to the potential development of severe nosocomial infections^41^. Therefore, preventing hospitalization of any kind is essential.

The protective effect of SGLT-2 inhibitors against hospitalization found in our study perhaps rests upon the fact that SGLT-2 inhibitors show cardioprotective effects as a class. Extensive clinical trials found that they significantly reduced the relative risk of cardiovascular death and hospitalization for heart failure in patients with type 2 diabetes plus cardiovascular disease and that they decreased the risk of heart failure in type 2 diabetes mellitus patients with and without a cardiovascular disease history in routine care^42–44^. Their effect seems to be independent of the glycaemic status of the patient, in several clinical trials showing a general cardio-protective effect^45^. There are several other possible mechanisms which explain beneficial effects of SGLT-2 inhibitors in COVID-19. COVID-19 infection can make anaerobic environment and increasing the production of lactate which causes cellular damage. Dapagliflozin, a SGLT-2 inhibitor, may reduce lactate concentration by increasing glucose utility in aerobic pathway and by increasing the urinary excretion of lactate^35^. SGLT-2 inhibitors can also exert anti-inflammatory effects, both on systemic and peripheral tissue through reduction in adipose-tissue inflammation which is characterized by weight loss. They also promote increased fat utilization, reduce obesity-induced inflammation, and reduce insulin resistance through activation of M2 macrophages. Adipose tissue itself plays an important role in the pathogenesis of cytokine storm in COVID-19^35^. In addition, SGLT-2 inhibitors are able to reduce the inflammatory response directly by inhibiting several pro-inflammatory cytokines such as IL-6 and TNF-alpha. These cytokines are closely related to high mortality from COVID-19^35^.Our study has several strengths and several limitations. We described the entire population of PDMC of the Republic of Croatia and not just a sample. Also, we provided information regarding other comorbidities that could affect COVID-19 outcomes, such as chronic obstructive pulmonary disease and renal disease. The limitations of the study are retrospective and observational design. Further on, part of the population was excluded from the analysis due to no medication data. Also, HbA1c and BMI data could not be included in logistic regression models due to insufficient data. Low availability of HbA1c and BMI data can to a certain extent be explained by the COVID-19 pandemic which has had a negative effect on the utilization of healthcare by diabetes patients. In Croatia, the number of diabetes panels (one of the sources of HbA1c and BMI data) had a sharp decrease in 2020 (from 102 087 in 2019 to 85 006 in 2020). A similar trend was observed regarding the numbers of visits to primary healthcare providers for diabetes-related problems and diabetes patients who visited their primary healthcare provider (from 3,611,506 visits in 2019 to 3,531,499 in 2020)^46^.

Further on, cardiomyopathy was defined as presence of ICD-10 code I42 in the system. However, cardiomyopathy may represent in patients with ischemic heart disease or hypertension so there is a possible overlap between these disorders. Since we do not use patients’ medical history but data from a public health registries, we could not determine the etiology of cardiomyopathy. Still, we considered that regardless of the cause of cardiomyopathy, code I42 will be present in the system for most patients suffering from cardiomyopathy.

Another limitiation of the study is that the information on severity of cardiomyopathy is lacking. Lastly, data analyzed were collected during 2020 and 2021, when the original SARS-CoV-2 was still dominant. Therefore our analysis results may not be applied to other SARS-CoV-2 variants.

## Conclusion

PDMC are at greater risk of acquiring SARS-CoV-2 infection, being hospitalized for COVID-19, and dying from COVID-19 compared to the entire diabetic population. SGLT-2 inhibitors therapy was a protective factor against SARS-CoV-2 infection and against COVID-19 hospitalization while sulfonylurea and insulin therapies were COVID-19 hospitalization risk factors. Insulin therapy was also associated with increased COVID-19 death risk. The body of evidence for diabetes patients and the association between their anti-diabetic therapies and COVID-19 outcomes are piling up, while research is needed for patients who also suffer from cardiomyopathy.

### Supplementary Information

Supplementary Information 1.

Supplementary Information 2.

## Data Availability

A dataset is available upon reasonable request. Requests should be sent to the corresponding author.
